# Supplementary material for: A potent Henipavirus cross-neutralizing antibody reveals a dynamic fusion-triggering pattern of the G-tetramer
Source: Nat Commun. 2024 May 21;15:4330. doi: 10.1038/s41467-024-48601-w (PMC11109247; doi:10.1038/s41467-024-48601-w)

A potent Henipavirus cross-neutralizing antibody reveals a dynamic fusion-triggering pattern of the G-tetramer

Pengfei Fan[1*†], Mengmeng Sun[2*], Xinghai Zhang[3*], Huajun Zhang[3], Yujiao Liu[1], Yanfeng Yao[3], Ming Li[2], Ting Fang[1], Bingjie Sun[1], Zhengshan Chen[1], Xiangyang Chi[1], Li Chen[3,4], Cheng Peng[3], Zhen Chen[3], Guanying Zhang[1], Yi Ren[1], Zixuan Liu[1], Yaohui Li[1], Jianmin Li[1], Entao Li[2], Wuxiang Guan[3], Shanshan Li[2,5,6], Rui Gong[3†], Kaiming Zhang[2,5,6†], Changming Yu[1†], Sandra Chiu[2,7,8†]

[1]Laboratory of Advanced Biotechnology, Institute of Biotechnology, Beijing, China.

[2]Division of Life Sciences and Medicine, University of Science and Technology of China, Hefei, China.

[3]State Key Laboratory of Virology, Wuhan Institute of Virology, Center for Biosafety Mega-Science, Chinese Academy of Sciences, Wuhan, China.

[4]University of Chinese Academy of Sciences, Beijing, China.

[5]Center for Advanced Interdisciplinary Science and Biomedicine of IHM, MOE Key Laboratory for Cellular Dynamics, Division of Life Sciences and Medicine, University of Science and Technology of China, Hefei, China.

[6]Department of Urology, The First Affiliated Hospital of USTC, Division of Life Sciences and Medicine, University of Science and Technology of China, Hefei, China.

[7]Department of Laboratory Medicine, The First Affiliated Hospital of USTC, Division of Life Sciences and Medicine, University of Science and Technology of China, Hefei, China.

[8]Key Laboratory of Anhui Province for Emerging and Reemerging Infectious Diseases, Hefei, Anhui 230027, China.

[*]These authors contributed equally: Pengfei Fan, Mengmeng Sun, Xinghai Zhang.

[†]e-mail: fanpengfei93@163.com; gongr@wh.iov.cn; kmzhang@ustc.edu.cn; yuchangming@126.com; qiux@ustc.edu.cn.

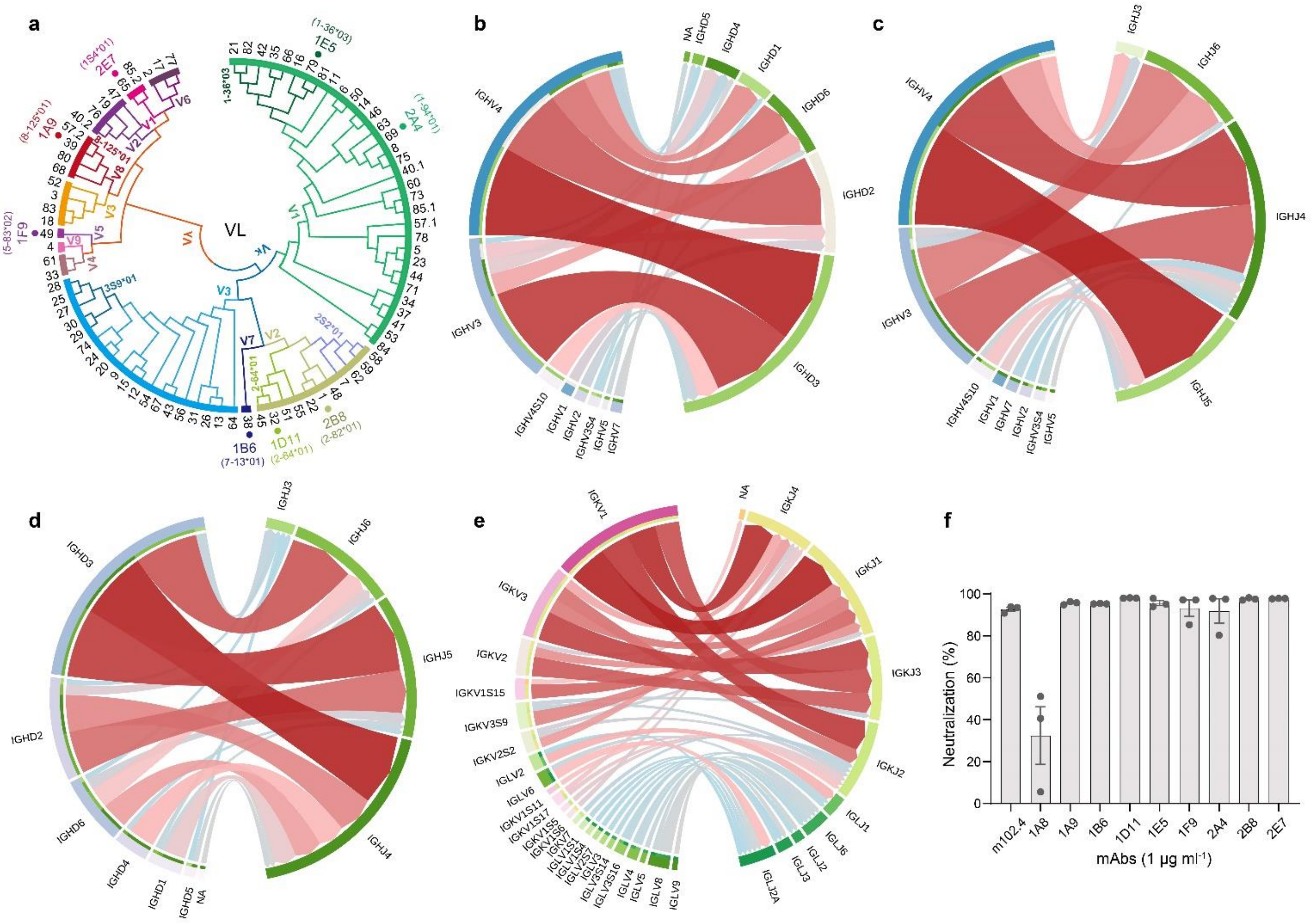

Supplementary Fig. 1 | Analysis of antibody-related germline genes. a Phylogeny of VL genes of functional antibodies. Genes encoding neutralizing antibodies or those with over four frequencies are marked. **b–e** Combinations of the V-D (**b**), V-J (**c**), or D-J (**d**) genes of the heavy chain or V-J (**e**) genes of the light chain of the NiV_{BD} G-specific antibodies. Each arc of the circle represents a family of V/D/J genes, and the width represents the frequency of the combination. **f** Neutralizing capacity of antibodies at 1 μg ml^{-1} against the rHIV-NiV_{BD} pseudovirus. Source data are provided as a Source Data file.

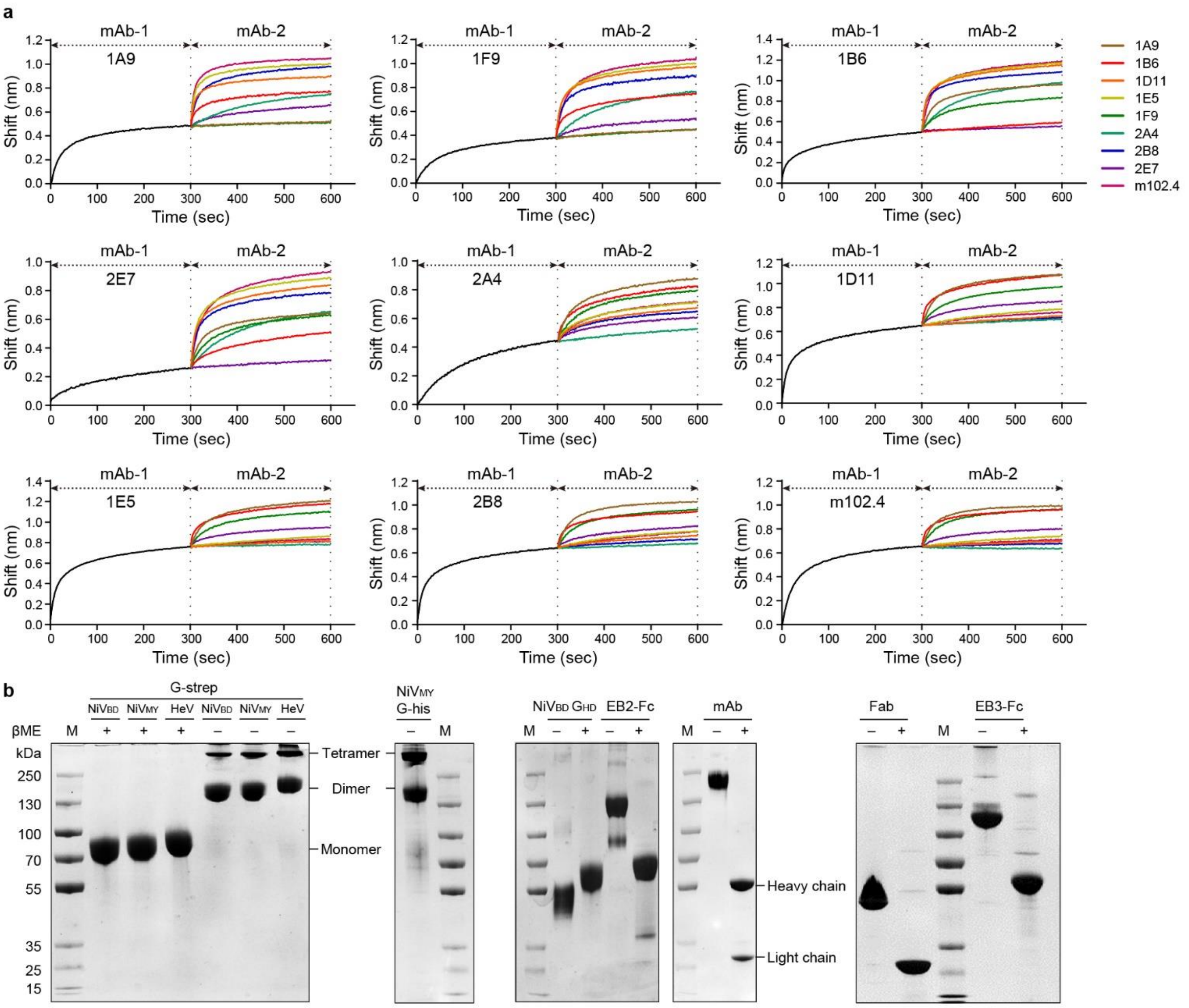

Supplementary Fig. 2 | Competition characteristics of antibodies and the production of proteins. a Competitive effects of neutralizing antibodies on binding to NiV_{BD} G. Gator™ streptavidin probes were first saturated with biotinylated NiV_{BD} G and then sequentially loaded with 100 nM primary antibody and 100 nM competing antibody. The binding percentage was calculated by comparing response values in the presence and absence of competing antibodies. **b** Protein production and SDS–PAGE analysis. The proteins were treated with non-denaturing (no β-ME, no boiling) or denaturing (β-ME, boiling) conditions and then loaded onto SurePAGE gels for electrophoresis. β-ME, β-mercaptoethanol; M, PageRuler Plus prestained protein ladder (Thermo Fisher Scientific). Purified G ectodomains were composed mainly of tetramers and dimers. The G head domain exists mainly as a monomer. The constructed ephrin B2/B3-Fc chimaeras formed a dimer under nonreducing conditions. Source data are provided as a Source Data file.

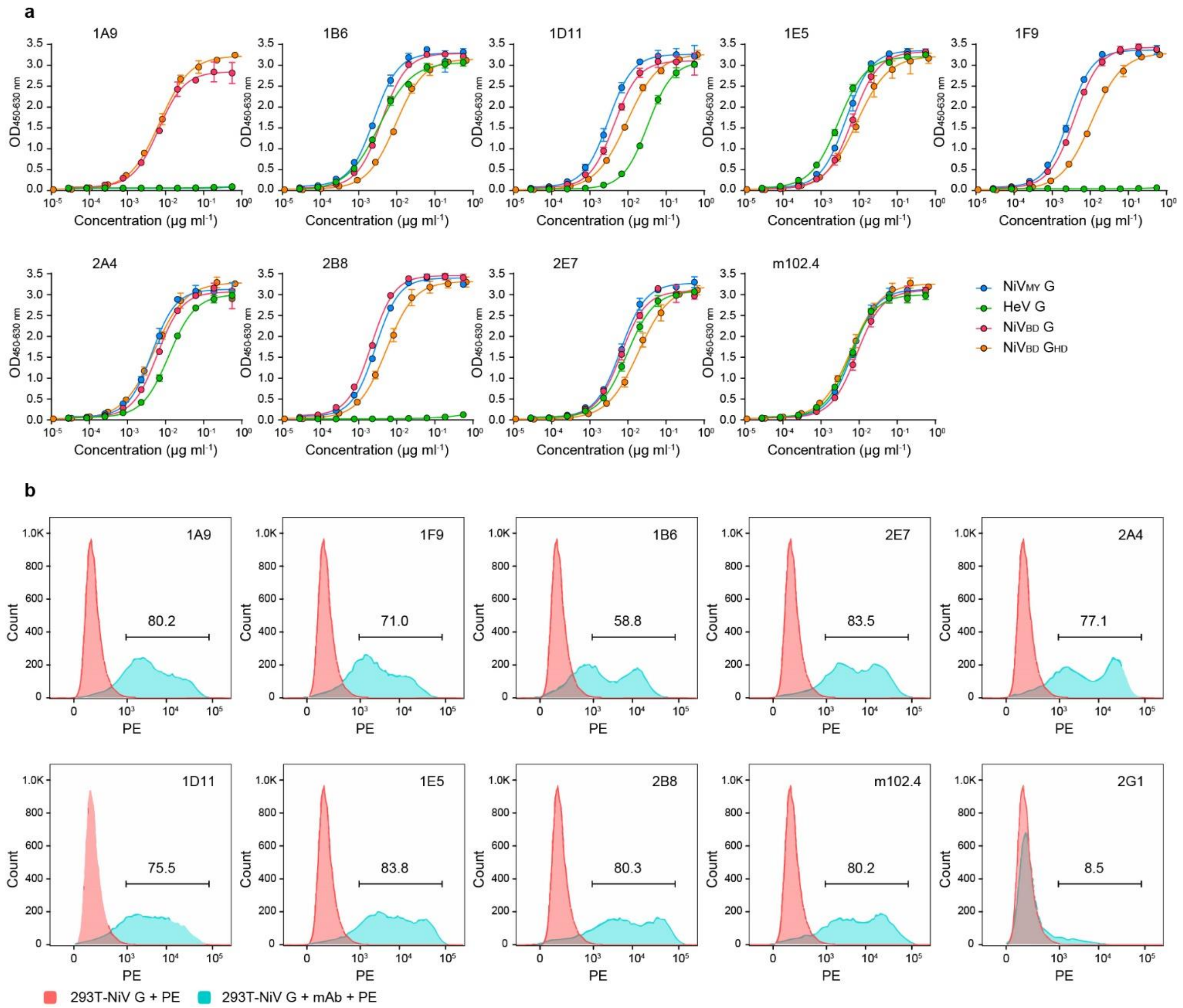

Supplementary Fig. 3 | The binding ability of antibodies to various forms of HNV G proteins. a Binding curves of antibodies to HNV G proteins. Binding of the antibody to four G proteins (NiV$_{BD}$ G, NiV$_{MY}$ G, HeV G, and NiV$_{BD}$ G$_{HD}$) was tested. The binding data were fitted to a four-parameter curve using GraphPad Prism software (version 8.0). Data are presented as the mean ± s.d. of one representative experiment. **b** Binding of antibodies to full-length NiV$_{BD}$ G on the 293T surface. NiV$_{BD}$ G was transiently expressed on the surface of 293T cells and incubated with specific antibodies or an isotype control IgG (2G1). The cells were stained with PE-conjugated mouse anti-human IgG, and the proportion of PE-positive cells among the total cells was analyzed using FlowJo V10 software. Data represent the means of triplicate measurements. Source data are provided as a Source Data file.

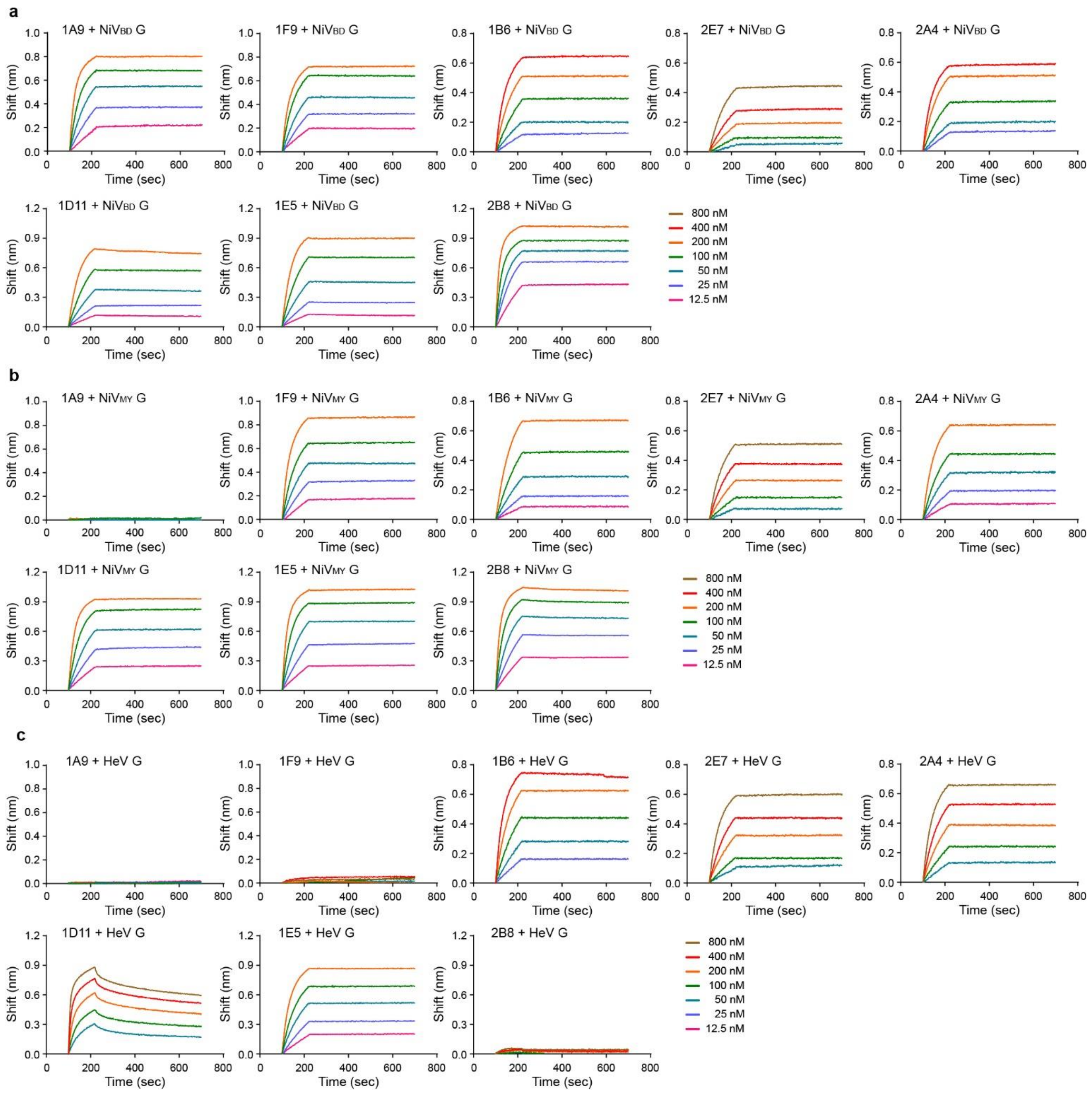

Supplementary Fig. 4 | The binding kinetics of eight nAbs to recombinant HNV G proteins. The antibodies were captured onto Gator™ Anti-Human Fc probes, and serial dilutions of NiV_{BD} (**a**), NiV_{MY} (**b**), or HeV (**c**) G proteins were then loaded. Five representative curves were fitted globally to a 1:1 Langmuir binding model using GatorTM Part 11 Software to calculate the affinity constants. Source data are provided as a Source Data file.

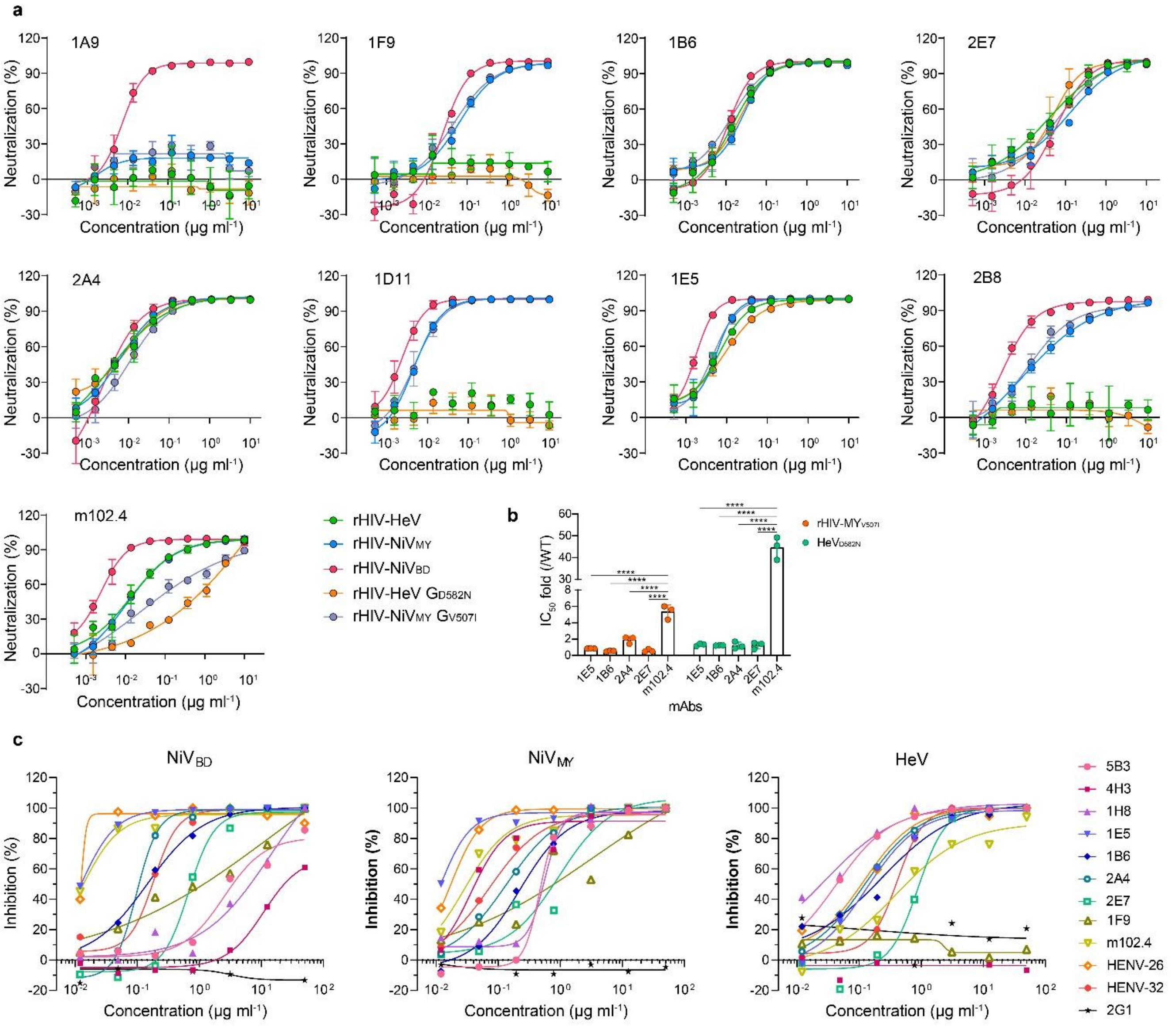

	5B3	4H3	1H8	1E5	1B6	2A4	2E7	1F9	m102.4	HENV-26	HENV-32	2G1
NiV-BD	3.487	23.427	6.685	0.012	0.113	0.107	0.741	1.188	0.014	0.012	0.177	
NiV-MY	0.496	0.043	0.539	0.012	0.255	0.137	0.791	0.800	0.032	0.017	0.080	
HeV	0.018		0.025	0.147	0.187	0.124	0.937		0.574	0.110	0.395	

Supplementary Fig. 5 | Neutralizing capacity of antibodies against HIV-pseudotyped or authentic HNVs. a HIV-pseudotyped HNVs were incubated with serially diluted mAbs and subsequently added to 293T cells. The percentage neutralization was calculated as the ratio of the reduced fluorescence intensity in the mAb-treated wells to that in the wells containing the virus only. Data are shown as the mean ± s.d. of three replicates from a representative experiment. **b** Changes in the neutralizing potency of bnAbs against rHIV-HNVs variants compared to the wild type. Data are shown as the mean ± s.d. of three replicates. Dunnett's multiple comparisons test was used, $****P < 0.0001$. **c** Evaluation of the neutralizing activity of representative G or F antibodies against live viruses. The IC_{50} values of the antibodies against HNVs were calculated and listed in the table. Source data are provided as a Source Data file.

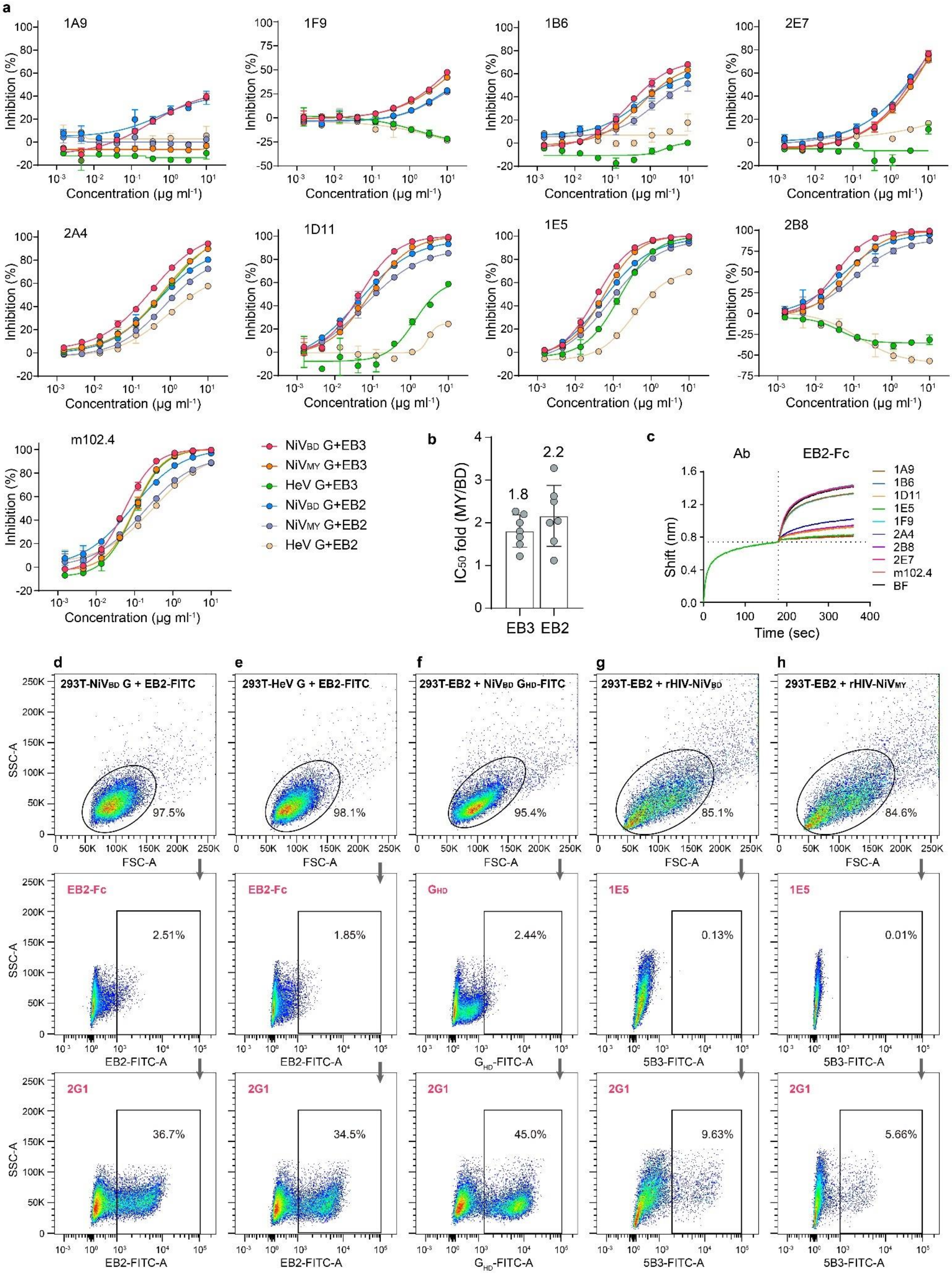

Supplementary Fig. 6 | Receptor blocking ability of the antibodies. a, b The ability of neutralizing antibodies to block receptor binding was assessed using Luminex assays. The data represent the average values of two replicates. The bar graph (**b**) shows the IC_{50} ratio of the antibody blocking receptor binding

to the two NiV strains. **c,** The ability of antibodies to block the binding of recombinant receptors to soluble G ectodomains tested via BLI assays. **d-h** Images showing the gating strategy for each flow analysis experiment related to Fig. 3c–g, respectively. All FACS were gated using positive and negative controls, and the same strategy was applied to one experiment. Source data are provided as a Source Data file.

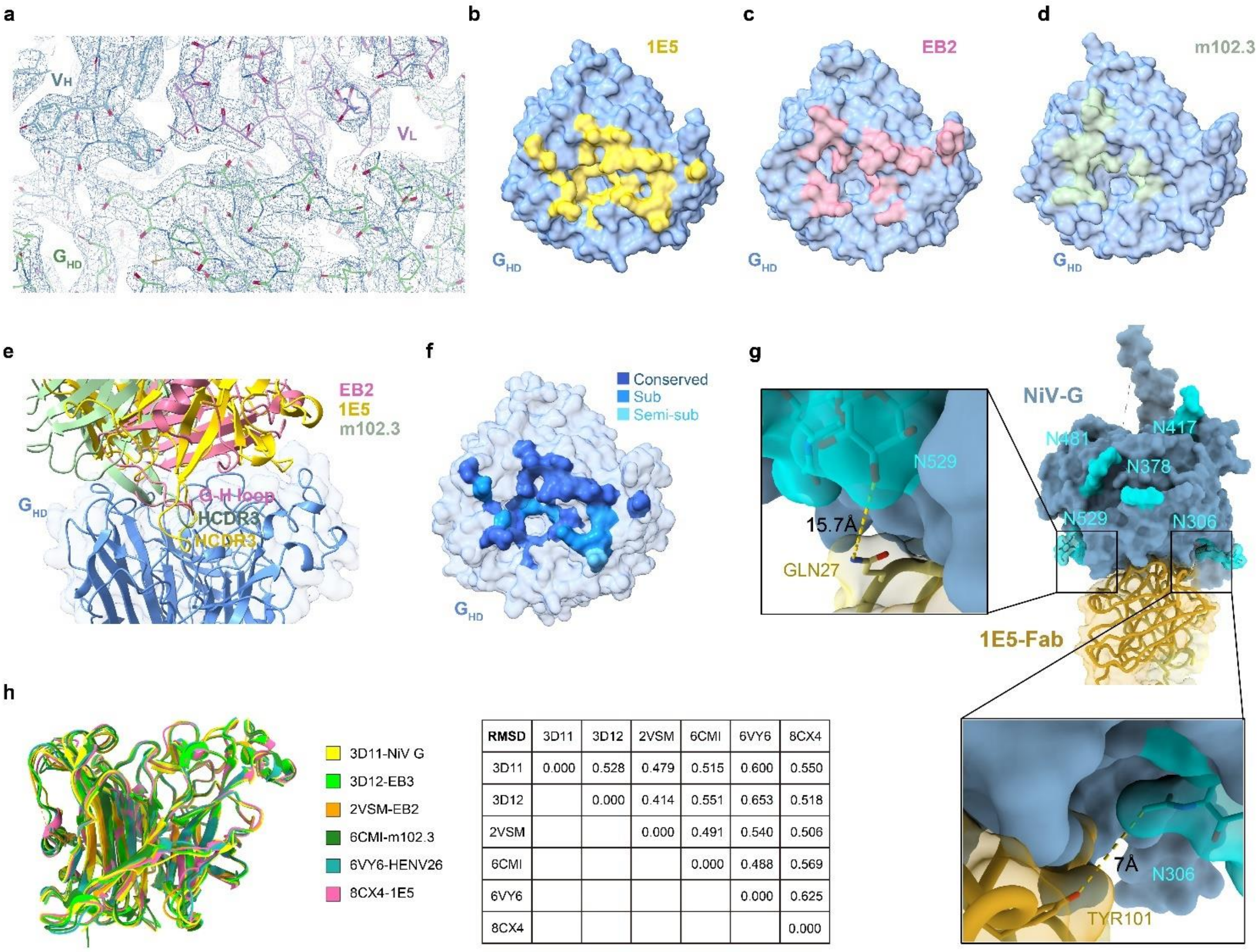

RMSD	3D11	3D12	2VSM	6CMI	6VY6	8CX4
3D11	0.000	0.528	0.479	0.515	0.600	0.550
3D12		0.000	0.414	0.551	0.653	0.518
2VSM			0.000	0.491	0.540	0.506
6CMI				0.000	0.488	0.569
6VY6					0.000	0.625
8CX4						0.000

Supplementary Fig. 7 | Structural analysis of G_{HD}-related complexes. a In the crystal form ($P4_12_12$), a portion of the interface of G_{HD} (green) and 1E5 Fab (V_H in cadet blue and V_L in purple) was displayed with electron density calculated using 2Fo-Fc coefficients and contoured at 1.2 σ as a blue mesh. **b-d** Epitope imprints of 1E5 (**b**), EB2 (**c**), and m102.3 (**d**) on the G head. **e** Zoomed-in views of the NiV G interface in complex with 1E5, EB2, or m102.3. **f** Binding site mapping of 1E5 onto the NiV G_{HD} or HeV G_{HD} (PDB ID: 2VSK) surfaces. The conserved, sub (conservative substitution), and semi-sub (semi-conservative substitution) regions are colored as indicated. **g** The impact of N-glycan on the binding of 1E5 to G. The NiV G-protomer/1E5 complex is shown as the molecular surface. The dark turquoise indicates N-glycans. The distance between the 1E5 Fab and N-linked glycosylation at position N306/N529 is shown as a gold dotted line. **h** The superimposition of G-heads determined by receptors or RBD-targeting antibodies with the unbound G-head. The RMSD matrix between pairwise structures is shown in the right table.

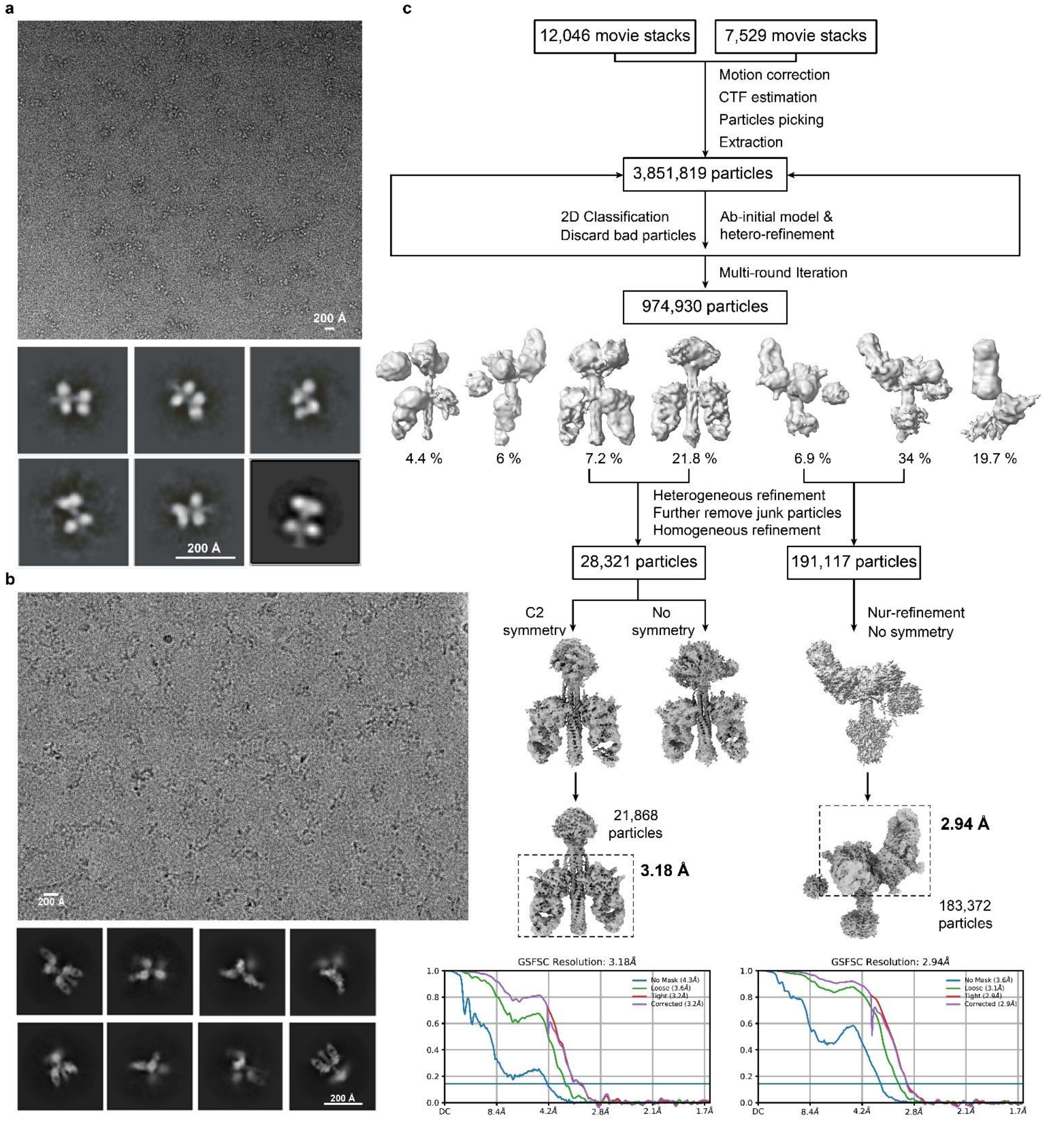

Supplementary Fig. 8 | Negative-stain and cryo-electron microscopy data collection and processing. **a** Verification of the structural correctness of the NiV_{MY} G ectodomain using negative-stain EM. Scale bar: 200 Å. **b** Representative motion-corrected cryo-EM micrographs and reference-free 2D class averages. Scale bar: 200 Å. **c** Workflow of the data processing procedure.

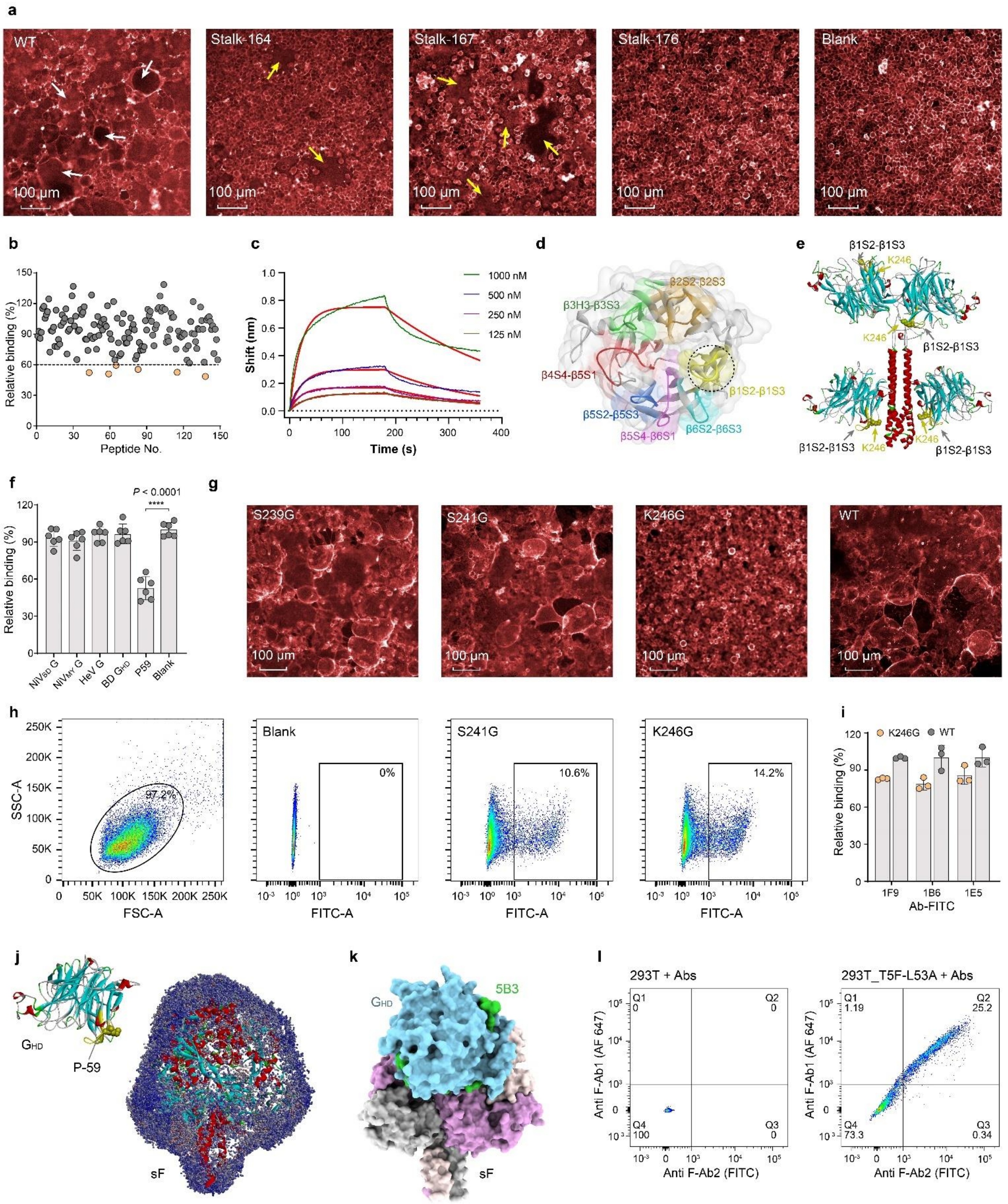

Supplementary Fig. 9 | Identification of critical sites involved in G-F interactions. a Fusion of 293T cells induced by cotransfection of NiV T5F with full-length or headless NiV G. White arrows indicate typical syncytia and yellow arrows indicate suspected shattered areas. The experiments were performed twice, and similar results were obtained. **b** Peptides that interact with sF were screened using nickel-coated plates and overlapping peptides spanning the NiV_{BD} G. The relative binding of h5B3.1 at the EC_{50} was

calculated. **c** Affinity between P-59 and sF tested by BLI. The experiments were performed twice, and similar results were obtained. **d** The amino acids corresponding to P-59 form the extended outer loop β1S2-β1S3 (black dotted circle) located on the edge of the central cavity. **e** The location of P-59 in the G-tetramer. **f** Analysis of the interaction between soluble HNV G and sF. Data are six replicates from one test. **g** Representative images of 293T cell–cell fusion induced by NiV T5F with wild-type or mutant NiV G. The experiments were performed three times, and similar results were obtained. **h** Representative images of the binding of EB2 to the wild-type or mutant G expressed on the 293T cell surface (related to Fig. 6d). **i** Relative binding of nAbs from different groups to the full-length K246G variant of G displayed on 293T cells. Data are the mean ± s.d. of three replicates. **j** All docked poses of G_{HD} and sF generated by Discovery Studio 4.5. **k** The superimposition of the top 1 pose of G_{HD}-sF with the G_{HD}-5B3 structure. Molecules are shown in colored surface representation. **l** Representative images of two noncompetitive F-specific antibodies binding to surface-displayed T5F wild-type or mutants (related to Fig. 6i). Source data are provided as a Source Data file.

Supplementary Table 1 | Summary of antibody binding kinetics.

Ligand	Analyte	K_{on} (M^{-1} s^{-1})	k_{on} Error	K_{off} (s^{-1})	k_{off} Error	K_D (M)	Full R^2	Full χ^2
1A9		4.82E+05	1.33E+03	8.37E-06	1.35E-06	1.74E-11	0.992	2.2
1F9		1.36E+05	3.92E+02	1.60E-06	1.28E-06	1.18E-11	0.997	1.7
1B6		5.00E+04	1.73E+02	<1.00E-06	NA	<2.00E-11	0.997	1.1
2E7	NiV_{BD}	1.03E+04	8.68E+01	<1.00E-06	NA	<9.71E-11	0.997	0.8
2A4	G	5.28E+04	1.89E+02	<1.00E-06	NA	<1.89E-11	0.997	1.3
1D11		2.08E+05	6.02E+02	8.92E-05	1.25E-06	4.28E-10	0.998	1.4
1E5		2.33E+05	6.10E+02	1.26E-05	1.17E-06	5.40E-11	0.999	1.8
2B8		3.31E+05	9.17E+02	<1.00E-06	NA	<3.02E-12	0.996	2.1
1A9		NA	NA	NA	NA	NA	NA	NA
1F9		1.29E+05	3.63E+02	<1.00E-06	NA	<7.75E-12	0.998	2.0
1B6		6.28E+04	2.52E+02	<1.00E-06	NA	<1.59E-11	0.999	0.9
2E7	NiV_{MY}	2.02E+04	7.31E+01	4.80E-06	1.30E-06	2.38E-10	0.998	0.7
2A4	G	8.78E+04	2.82E+02	<1.00E-06	NA	<1.14E-11	0.998	0.9
1D11		3.27E+05	8.74E+02	<1.00E-06	NA	<3.06E-12	0.997	2.7
1E5		3.39E+05	9.34E+02	<1.00E-06	NA	<2.95E-12	0.996	2.7
2B8		2.76E+05	7.58E+02	1.47E-05	1.28E-06	5.31E-11	0.997	1.9
1A9		NA	NA	NA	NA	NA	NA	NA
1F9		NA	NA	NA	NA	NA	NA	NA
1B6		7.09E+04	1.90E+02	3.97E-05	1.25E-06	5.60E-10	0.998	1.6
2E7	HeV G	2.53E+04	8.17E+01	<1.00E-06	NA	<3.95E-11	0.998	1.0
2A4		2.98E+04	7.75E+01	2.33E-06	8.37E-07	7.84E-11	0.998	1.2
1D11		2.20E+05	1.37E+03	8.03E-04	3.57E-06	3.66E-09	0.998	1.1
1E5		1.50E+05	6.09E+02	<1.00E-06	NA	<6.67E-12	0.998	1.1
2B8		NA	NA	NA	NA	NA	NA	NA

Four or five representative curves were used to calculate the affinity constant and obtain better goodness of fit (full R^2 >0.99 and full χ^2 <3). NA, not available; kon, association rate; koff, dissociation rate; K_D, affinity constant.

Supplementary Table 2 | Crystal data collection and refinement statistics.

	NiV G_{HD}-1E5 Fab (PDB ID: 8XC4)
**Data collection**	
Wavelength (Å)	0.979
Resolution (Å)	50-3.26 (3.32-3.26)
Space group	P 4_12_12
Cell dimensions	193.4 193.4 198.1
(Å/degree)	90.0 90.0 90.0
Unique reflections	58,116 (2,888)
Completeness (%)	97.1 (97.9)
Rmeas (%)	20.6 (133.7)
Rpim (%)	7.8 (51.1)
Redundancy	6.4 (6.1)
Average $I/\sigma(I)$	9.6 (1.1)
Wilson B value (Å^2)	89.20
**Statistics for Refinement**	
Resolution (Å)	32.32-3.24 (3.36-3.24)
Rwork (%)	22.1 (31.7)
Rfree (%)	24.7 (32.7)
Reflections used	57,368 (5,445)
R.m.s.d.	
Bond (degree)	1.200
Length (Å)	0.006
No. of atoms	13,778
proteins	13,444
Average B factors (Å^2)	110.6
proteins	109.5
Ramachandran plot	
Favored region (%)	94.78
Allowed region (%)	5.22
Outliers (%)	0.00

Supplementary Table 3 | Cryo-EM data collection, refinement, and validation statistics.

	#1 NiV G/1E5 compact type (EMDB-36760, PDB 8K0C)	#2 NiV G/1E5 loose type (EMDB-36761, PDB 8K0D)
Data collection and processing		
Magnification	105,000	
Voltage (kV)	300	
Electron exposure (e–/Å^2)	51	
Defocus range (μm)	1.6–3.2	
Pixel size (Å)	0.82	
Symmetry imposed	C2	C1
Initial particle images (no.)	1,109,405	
Final particle images (no.)	21,868	183,372
Map resolution (Å)	3.18	2.94
FSC threshold	0.143	0.143
Refinement		
Initial model used (PDB code)	7TY0	8K0C
Model resolution (Å)	3.18	2.94
FSC threshold	0.143	0.143
Model composition		
Non-hydrogen atoms	15,262	13,282
Protein residues	1986	1714
R.m.s. deviations		
Bond lengths (Å)	0	0
Bond angles (°)	0	0.01
Validation		
MolProbity score	1.69	1.94
Clashscore	5.51	12.59
Poor rotamers (%)	0	0.07
Ramachandran plot		
Favored (%)	94.2	95.3
Allowed (%)	5.7	4.58
Disallowed (%)	0.1	0.12

Source data of all gels presented in Supplementary Figure 2b are supplied as follows.

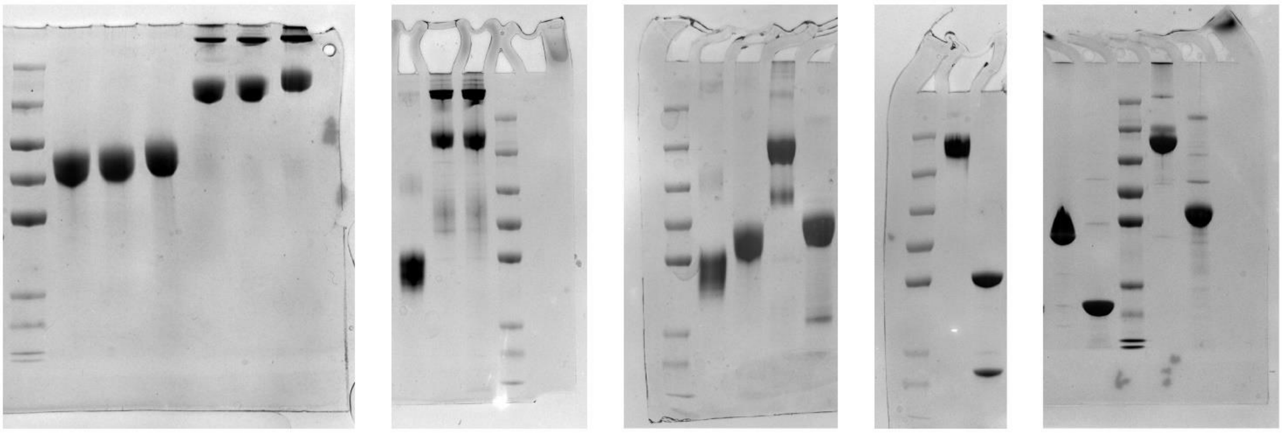

Supplement: Supplementary file 1 — Supplementary Information file [file 41467_2024_48601_MOESM1_ESM.pdf]
