# Supplementary material for: A potent Henipavirus cross-neutralizing antibody reveals a dynamic fusion-triggering pattern of the G-tetramer
Source: Nat Commun. 2024 May 21;15:4330. doi: 10.1038/s41467-024-48601-w (PMC11109247; doi:10.1038/s41467-024-48601-w)
Supplement: Supplementary file 3 — Reporting Summary [file 41467_2024_48601_MOESM3_ESM.pdf]

nature portfolio

Corresponding author(s): Pengfei Fan, Rui Gong, Kaiming Zhang, Changming Yu, Sandra Chiu

Last updated by author(s): Apr 26, 2024

Reporting Summary

Nature Portfolio wishes to improve the reproducibility of the work that we publish. This form provides structure for consistency and transparency in reporting. For further information on Nature Portfolio policies, see our Editorial Policies and the Editorial Policy Checklist.

Statistics

For all statistical analyses, confirm that the following items are present in the figure legend, table legend, main text, or Methods section.

n/a	Confirmed	
☐	☒	The exact sample size (n) for each experimental group/condition, given as a discrete number and unit of measurement
☐	☒	A statement on whether measurements were taken from distinct samples or whether the same sample was measured repeatedly
☐	☒	The statistical test(s) used AND whether they are one- or two-sided<br>*Only common tests should be described solely by name; describe more complex techniques in the Methods section.*
☒	☐	A description of all covariates tested
☒	☐	A description of any assumptions or corrections, such as tests of normality and adjustment for multiple comparisons
☐	☒	A full description of the statistical parameters including central tendency (e.g. means) or other basic estimates (e.g. regression coefficient) AND variation (e.g. standard deviation) or associated estimates of uncertainty (e.g. confidence intervals)
☐	☒	For null hypothesis testing, the test statistic (e.g. F, t, r) with confidence intervals, effect sizes, degrees of freedom and P value noted<br>*Give P values as exact values whenever suitable.*
☒	☐	For Bayesian analysis, information on the choice of priors and Markov chain Monte Carlo settings
☒	☐	For hierarchical and complex designs, identification of the appropriate level for tests and full reporting of outcomes
☒	☐	Estimates of effect sizes (e.g. Cohen's d, Pearson's r), indicating how they were calculated

Our web collection on statistics for biologists contains articles on many of the points above.

Software and code

Policy information about availability of computer code

Data collection

As described in Methods:
* Protein sequences were obtained from GenBank (http://www.ncbi.nlm.nih.gov/genbank/).
* Protein purification monitored by AKTA pure chromatography system using UNICORN software version 7.0 (GE Healthcare).
* B cells were sorted using a MoFlo XDP flow cytometer (Beckman Coulter) with Summit Software version 5.1.3.
* BLI data were collected using Gator Bioanalysis System (GatorBio) with GatorLaunch version 2.10.4.0713.
* FACS data were collected by a FACSCanto II flow cytometer (BD Biosciences) using BD FACSDiva™ software v8.0.1.
* ELISA data were obtained using SpectraMax 190 Microplate Reader (Molecular Devices) with SoftMax Pro v6.3.
* Neutralization data were read by a Spark multimode microplate reader (TECAN) using SparkControl v1.2.
* Luminex data were collected using MAGPIX Multiplexing System (Luminex) with MAGPIX® xPONENT® 4.3.
* Fusion images were captured using Operetta CLS High Content Analysis System (PerkinElmer) with Harmony 4.9.
* Cryo-EM data were acquired by a Titan Krios G3i and EPU 2.12 (ThermoFisher Scientific).
* MST data were collected using Monolith NT.115 (NanoTemper Tech).

Data analysis

* ELISA, neutralization, and Luminex assays were analyzed using GraphPad Prism version 8.0.
* Sequence analysis: IMGT/V-QUEST (https://www.imgt.org/IMGT_vquest), ClustalW (https://www.genome.jp/tools-bin/clustalw), MEGA version 7.0.14, Circlize v0.4.12, and Vector NTI Advance 11.5.1.
* PCR products were analyzed with the QIAxcel Advanced system (QIAGEN) using QIAxcel ScreenGel 1.6.0.
* FACS data analyzed using FlowJo version 10 (Tree Star Inc.).
* BLI data analyzed with GatorTM Part11 Software (GatorBio) using GatorLaunch version 2.10.4.0713.

* Neutralization plaques and cell fusion areas were analyzed using ImageJ 1.52a.
* Crystallization data were processed using HKL2000, Phenix 1.13, Coot 0.9.2, and PyMOL 2.4.0.
* Cryo-EM data were processed using CryoSPARC3.2.0 and analyzed with SWISS-MODEL, Alphafold2-multimer, MDFF, Coot 0.9.2, ChimeraX 1.6, Phenix 1.13, and MolProbity.
* Figures were arranged in Adobe Illustrator CC 2022.

For manuscripts utilizing custom algorithms or software that are central to the research but not yet described in published literature, software must be made available to editors and reviewers. We strongly encourage code deposition in a community repository (e.g. GitHub). See the Nature Portfolio guidelines for submitting code & software for further information.

Data

Policy information about availability of data

All manuscripts must include a data availability statement. This statement should provide the following information, where applicable:

- Accession codes, unique identifiers, or web links for publicly available datasets
- A description of any restrictions on data availability
- For clinical datasets or third party data, please ensure that the statement adheres to our policy

The crystal structure of 1E5-Fab complexed with the NiVBD GHD and the cryo-EM structures of 1E5-Fab complexed with the NiVMY G-tetramer have been deposited in the Protein Data Bank and Electron Microscopy Data Bank (EMDB) with the accession codes PDB ID 8XC4 (https://doi.org/10.2210/pdb8XC4/pdb), PDB ID 8K0C (to be released), PDB ID 8K0D (to be released), EMDB-36760 (to be released), and EMDB-36761 (to be released). This study also used 7TXZ (https://doi.org/10.2210/pdb7TXZ/pdb), 7TY0 (https://doi.org/10.2210/pdb7TY0/pdb), 2VWD (https://doi.org/10.2210/pdb2VWD/pdb), 2VSM (https://doi.org/10.2210/pdb2VSM/pdb), 2VSK (https://doi.org/10.2210/pdb2VSK/pdb), 3D11 (https://doi.org/10.2210/pdb3D11/pdb), 3D12 (https://doi.org/10.2210/pdb3D12/pdb), 6CMI (https://doi.org/10.2210/pdb6CMI/pdb), 6TYS (https://doi.org/10.2210/pdb6TYS/pdb), and 6VY6 (https://doi.org/10.2210/pdb6VY6/pdb) from the Protein Data Bank. Further requests for materials should be addressed to S.C. (qiux@ustc.edu.cn). Source data are provided with this paper.

Research involving human participants, their data, or biological material

Policy information about studies with human participants or human data. See also policy information about sex, gender (identity/presentation), and sexual orientation and race, ethnicity and racism.

Reporting on sex and gender	No human participants or human data were involved in this study.
Reporting on race, ethnicity, or other socially relevant groupings	No human participants or human data were involved in this study.
Population characteristics	No human participants or human data were involved in this study.
Recruitment	No human participants or human data were involved in this study.
Ethics oversight	No human participants or human data were involved in this study.

Note that full information on the approval of the study protocol must also be provided in the manuscript.

Field-specific reporting

Please select the one below that is the best fit for your research. If you are not sure, read the appropriate sections before making your selection.

☒ Life sciences ☐ Behavioural & social sciences ☐ Ecological, evolutionary & environmental sciences

For a reference copy of the document with all sections, see nature.com/documents/nr-reporting-summary-flat.pdf

Life sciences study design

All studies must disclose on these points even when the disclosure is negative.

Sample size	All in vitro molecule biology or cell experiments were conducted in triplicate following standard practices. In virus challenge experiments, six biologically independent animals for each group were determined, similar to those generally employed in the field (PMID: 34469726; PMID: 38582870; PMID: 37925490). Due to the limited resource of BSL-4 laboratory, only two animal experiments were conducted.
Data exclusions	No data was excluded.
Replication	In vitro assays were performed in 2-3 independent. In vivo protection test was performed with six hamsters per experiment group. All attempts at replication were successful.
Randomization	The allocation into experimental groups was random in all animal experiments.
Blinding	Blinding was not a relevant feature in this study. This is a non-clinical study with data collection and analyses relying on objective measures.

Reporting for specific materials, systems and methods

We require information from authors about some types of materials, experimental systems and methods used in many studies. Here, indicate whether each material, system or method listed is relevant to your study. If you are not sure if a list item applies to your research, read the appropriate section before selecting a response.

Materials & experimental systems

n/a	Involved in the study
☐	☒ Antibodies
☐	☒ Eukaryotic cell lines
☒	☐ Palaeontology and archaeology
☐	☒ Animals and other organisms
☒	☐ Clinical data
☒	☐ Dual use research of concern
☒	☐ Plants

Methods

n/a	Involved in the study
☒	☐ ChIP-seq
☐	☒ Flow cytometry
☒	☐ MRI-based neuroimaging

Antibodies

Antibodies used	The commercial antibodies used in the article are:<br>1. PerCP mouse anti-Hu/NHP CD3, BD Pharmingen, Cat.# 552851, Clone SP34-2, 10 μL/5×10^5cells;<br>2. APC mouse anti-human CD19, Beckman, Cat.# IM2470, Clone J3-119, 10 μL/5×10^5cells;<br>3. PE-Cy7 mouse anti-human, Beckman, Cat.# B49205, Clone 1A4CD27, 10 μL/5×10^5cells;<br>4. PE mouse anti-human IgG, BD Pharmingen, Cat.# 555787, Clone G18-145, 15 μL/5×10^5cells;<br>5. Goat Anti-Monkey IgG H&L (HRP), Abcam, Cat.# ab112767, 1:10000 dilution;<br>6. Goat Anti-Human IgG Fc (HRP), Abcam, Cat.# 97225, 1:10000 dilution.<br><br>The variable sequences of m102.4 (Patent No US14026142B2), h5B3.l (Patent No. US15951327B2), HENV-26 (Patent No. W02021097024), HENV-32 (Patent No. W02022132710), 4H3 (PMID: 36932063), and 1H8 (PMID: 36932063) were obtained, synthesized and constructed into the constant region of human lgGl before production.<br><br>The remaining G or F antibodies involved in the article were developed by the research team.
Validation	All the fluorescent or secondary antibodies were bought from commercial vendors and were validated by the manufacturers:<br>1. PerCP mouse anti-Hu/NHP CD3 (https://www.bdbiosciences.com/zh-cn/products/reagents/flow-cytometry-reagents/research-reagents/single-color-antibodies-ruo/percp-mouse-anti-human-cd3.552851);<br>2. APC mouse anti-human CD19 (https://www.mybeckman.cn/reagents/coulter-flow-cytometry/antibodies-and-kits/single-color-antibodies/cd19/im2470);<br>3. PE-Cy7 mouse anti-human (https://www.mybeckman.cn/reagents/coulter-flow-cytometry/antibodies-and-kits/single-color-antibodies/cd27/b49205);<br>4. PE mouse anti-human IgG (https://www.bdbiosciences.com/zh-cn/products/reagents/flow-cytometry-reagents/research-reagents/single-color-antibodies-ruo/pe-mouse-anti-human-igg.555787);<br>5. Goat Anti-Monkey IgG H&L (HRP) (https://www.abcam.cn/products/secondary-antibodies/goat-monkey-igg-hl-hrp-ab112767.html);<br>6. Goat Anti-Human IgG Fc (HRP) (https://www.abcam.cn/products/secondary-antibodies/goat-human-igg-fc-hrp-ab97225.html).<br><br>Recombinant antibodies m102.4 (PMID: 18271743), h5B3.l (PMID: 22915804), HENV-26 (PMID: 33306954), HENV-32 (PMID: 33306954), 4H3 (PMID: 36932063), and 1H8 (PMID: 36932063) were reported and verified in previous studies.

Eukaryotic cell lines

Policy information about cell lines and Sex and Gender in Research

Cell line source(s)	HEK293T (CRL-11268) and Vero E6 (CRL-1586) cells were obtained from ATCC, and Expi293F (A14528) cells were obtained from ThermoFisher Scientific.
Authentication	None of the cell lines used here were authenticated.
Mycoplasma contamination	All used cell stocks tested negative for mycoplasma.
Commonly misidentified lines<br>(See ICLAC register)	None of the commonly misidentified cell lines were used in the study

Animals and other research organisms

Policy information about studies involving animals; ARRIVE guidelines recommended for reporting animal research, and Sex and Gender in Research

Laboratory animals	A rhesus macaque (Macaca mulatta), female, 5 years old; Golden hamsters (Mesocricetus auratus), female, 4-5 weeks old, 100 g.
Wild animals	The study did not involve wild animals.
Reporting on sex	Sex of the animals is not considered relevant for the studies.
Field-collected samples	The study did not involve samples collected from the field.
Ethics oversight	The Institutional Animal Care and Use Committee of laboratory Animal Center of Academy of Military Medical Sciences approved rhesus macaque studies (approval no. IACUC-DWZX-2020-052); The Life Science Ethics Committee of the Wuhan Institute of Virology approved hamster studies (approval no. WIVA45202105).

Note that full information on the approval of the study protocol must also be provided in the manuscript.

Plants

Seed stocks	Plant materials were not used in this study.
Novel plant genotypes	Plant materials were not used in this study.
Authentication	Plant materials were not used in this study.

Flow Cytometry

Plots

Confirm that:

- [x] The axis labels state the marker and fluorochrome used (e.g. CD4-FITC).
- [x] The axis scales are clearly visible. Include numbers along axes only for bottom left plot of group (a 'group' is an analysis of identical markers).
- [x] All plots are contour plots with outliers or pseudocolor plots.
- [x] A numerical value for number of cells or percentage (with statistics) is provided.

Methodology

Sample preparation	For cell sorting, fresh monkey blood was diluted with PBS and then added to the upper layer of the monkey lymphocyte separation solution (Dakewe), followed by Ficoll-Hypaque density gradient centrifugation to obtain peripheral blood mononuclear cells. Single NiV G-specific memory B cells were stained and sorted as described in the manuscript.<br>For flow cytometric analysis, HEK293T cells were transiently transfected with genes of henipavirus glycoproteins or ephrin-B2 and digested with PBS containing 0.02% (w/v) EDTA, followed by wash and stain procedures.
Instrument	MoFlo XDP cell sorter (Beckman Coulter) for cell sorting; FACSCanto II flow cytometer (BD Biosciences) for flow cytometric analysis.
Software	Summit Software verison 5.1.3 for MoFlo XDP flow cytometer and BD FACSDiva Software v8.0.1 for FACSCanto II flow cytometer.
Cell population abundance	For cell sorting, relevant cell populations were provided in each gate.<br>For flow analysis, HEK293T cell line transiently transfected to express Ephrin-B2 or HNVs G, and the abundance of positive cells was affected by the protein property, cell viability, well/flask size, and experimental protocol.
Gating strategy	FSC/SSC gate was set to exclude dead/debris cells, and FSC-H/FSC-W gate was set to analyze single cells only. For cell sorting, PBMCs stained with single fluorescence were used to calculate compensation. For flow analysis, an irrelevant antibody was used to define the negative population.

- [x] Tick this box to confirm that a figure exemplifying the gating strategy is provided in the Supplementary Information.
